# Prescription of potentially inappropriate medication in Korean older adults based on 2012 Beers Criteria: a cross-sectional population based study

**DOI:** 10.1186/s12877-016-0285-3

**Published:** 2016-06-02

**Authors:** You-Seon Nam, Jong Soo Han, Ju Young Kim, Woo Kyung Bae, Kiheon Lee

**Affiliations:** Department of Family Medicine, Seoul National University Hospital, Seoul National University College of Medicine, Seoul, South Korea; Health Promotion Center, Seoul National University Bundang Hospital, Seoul National University College of Medicine, Seongnam, South Korea; Department of Family Medicine, Seoul National University Bundang Hospital, Seoul National University College of Medicine, Seongnam, South Korea

**Keywords:** Potentially inappropriate medication (PIM), Beers Criteria, Polypharmacy, Adverse drug effects (ADEs)

## Abstract

**Background:**

A high number of elderly people with multiple comorbidities are exposed to the risk of polypharmacy and prescription of potentially inappropriate medication (PIM). The purpose of this study was to determine the prevalence and patterns of PIM prescription in Korean older adults according to the 2012 Beers Criteria.

**Methods:**

A retrospective study was conducted using data from the Korean Health Insurance Review and Assessment (KHIRA) database of outpatient prescription claims collected from January 1, 2009 to December 31, 2011. A total of 523,811 elderly subjects aged 65 years and older were included in the study, and several covariates related to the prescription of PIMs were obtained from the KHIRA database. These covariates were analyzed using Student’s *t* test and the chi-square test; furthermore, multivariate logistic regression analysis was used to evaluate the risk factors associated with the prescription of PIMs.

**Results:**

A total of 80.96 % subjects were prescribed at least one PIM independent of their diagnosis or condition according to the 2012 Beers Criteria. The most commonly prescribed medication class was first-generation antihistamines with anticholinergic properties (52.33 %). Pain medications (43.04 %) and benzodiazepines (42.53 %) were next in line. When considering subjects’ diagnoses or conditions, subjects diagnosed with central nervous system conditions were most often prescribed PIMs. Female sex, severity of comorbidities, and polypharmacy were significant risk factors for PIM prescriptions.

**Conclusions:**

This study confirmed that PIM prescription is common among elderly Koreans. A clinical decision support system should be developed to decrease the prevalence of PIM prescriptions.

**Electronic supplementary material:**

The online version of this article (doi:10.1186/s12877-016-0285-3) contains supplementary material, which is available to authorized users.

## Background

As of 2011, elderly adults over 65 years old make up around 11.3 % of the Korean population. This is expected to continue to increase to 14 % by 2018, which would make the Korean population an “aged society” [1, 2]. Elderly adults are more likely to have more than one chronic illness or condition, which would require the concomitant prescription of several drugs. This makes them more vulnerable to the prescription of potentially inappropriate medications (PIMs), which can lead to an increased risk of adverse drug effects and unnecessary hospitalizations [3].

PIMs in older adults can be categorized into three groups: inappropriate medications regardless of comorbidities, medications that may exacerbate underlying diseases, and medications that may interact with other medications already in use [4]. Guidelines for PIMs have been developed in many countries, including the United States [5], Canada [4], France [6], Ireland [7], Australia [8], Norway [9], and South Korea [10]. Among these guidelines, the Beers Criteria, which were developed to be used as a guideline to avoid inappropriate prescribing in older adults, are the most commonly used explicit criteria for retrospective studies on the prescription rate of PIMs [11].

The Beers Criteria were initially developed and published by Beers and colleagues for nursing home residents in 1991 [12], and were subsequently expanded and revised in 1997 [13] and 2003 [14] to include all geriatric care settings. Then, an updated version was published in 2012 and was supported by the American Geriatrics Society. As a result, the 2012 Beers Criteria comprise fifty-three medications and medication classes divided into three categories: PIMs and classes to avoid in all older adults, PIMs and classes to avoid in older adults with certain diseases and syndromes that the drugs listed may exacerbate, and medications to be used with caution in older adults [5].

Researchers suggest that the PIMs specified in the Beers Criteria can exacerbate the condition and prognosis of older adults and have a negative influence on healthcare outcomes [3, 15–17]. In the case of the Korean population, clinicians have reported on the prevalence of PIMs in community-dwelling elderly [18], in older outpatients just before their admission to a general hospital [19], in a mixture of outpatients and inpatients in a general hospital [20], and in long-term care facilities [21]. However, no study has yet systematically examined PIM prescription rates in a population sample large enough to be representative of the entire Korean population.

The aim of this study was to determine the prevalence and patterns of PIM prescription in Korean older adults according to the 2012 Beers Criteria.

## Methods

### Data source

The study population was an assembly of three annual samples drawn from the Korean Health Insurance Review and Assessment (KHIRA) service database. It contains medical claims data for the entire Korean population as a result of the National Health Insurance System [22]. Each annual sample was randomly taken from 32 strata that were divided according to gender and age groups. Age groups were divided into sixteen 5-year age groups and subjects aged over 65 years were selected for this study. The sample size was 3 % of the entire Korean population.

The present study was submitted to the institutional review board of Seoul National University Bundang Hospital (reference number X-1307/209-904), and exempted from review because the data utilized for analysis was completely de-identified.

### Study population and medications

Subjects aged over 65 years at the time of a prescription, filling one or more prescription claim in 2009, 2010, or 2011 were included in the study. We excluded inpatients, and instead focused on outpatient prescriptions. The combined database for the three years contained information on 523,811 elderly subjects and a total of 45,727,527 prescriptions, from January 1, 2009, to December 31, 2011. When these prescriptions were examined separately by year, 19,165,885 prescriptions were from 2009; 13,201,065 were from 2010; and 13,360,577 were from 2011. Prescription information included the generic and trade names, prescription date, duration, and route of administration.

PIM prescription was assessed using the 2012 Beers Criteria [5]. We used two lists: one of individual medications or medication classes that are inappropriate for any patient aged 65 years and older, and the other of medications or medication classes that should be avoided for patients with certain diseases or syndromes. The list for medications to be used with caution in older adults was not included in the analysis because the data was based on claims data and the actual condition of the patients could not been assessed. In this study, we determined the prevalence of PIM prescriptions for these lists separately. All medications that were not available in Korea were excluded from the analysis.

### Covariates

Data concerning subjects’ age, gender, number of medications prescribed, types of healthcare facilities (long-term care, primary care, secondary care [which typically refers to large community but non-teaching hospitals], or tertiary care [which usually refers to a teaching or university hospital]) [22], residential area (urban or rural), type of insurance (national health insurance, medical aid, or veteran’s relief), and the number of outpatient department visits within one year were obtained from the database. Subjects were categorized by their age (65–69, 70–74, 75–79, 80–84, and ≥85), and the medical specialties that the subjects visited were classified into three groups: physicians, surgeons, and others. The specifics of each medical specialty subgroup are listed in Table 1. Subjects that had been prescribed at least one PIM, were divided into two subgroups. The first subgroup included subjects prescribed 1–4 PIMs, and the second subgroup included subjects prescribed ≥5 PIMs.

**Table 1:** Baseline characteristics (*N* = 523,811)

Characteristics		Male (*n* = 212,082) No (%)		Female (*n* = 311,729) No (%)		Total (*n* = 523,811) No (%)
Age, years						
65–69	85,169	(40.16)	101,098	(32.43)	186,267	(35.56)
70–74	62,964	(29.69)	85,631	(27.47)	148,595	(28.37)
75–79	37,059	(17.47)	62,664	(20.10)	99,723	(19.04)
80–84	17,559	(8.28)	38,087	(12.22)	55,646	(10.62)
≥85	9,331	(4.40)	24,249	(7.78)	33,580	(6.41)
No. of medications prescribed						
0–5	83,104	(39.18)	99,179	(31.82)	182,283	(34.80)
6–9	106,319	(50.13)	169,554	(54.39)	275,873	(52.67)
≥10	22,659	(10.68)	42,996	(13.79)	65,655	(12.53)
No. of medications from Beers Criteria 2012						
0	45,721	(21.56)	54,026	(17.33)	99,747	(19.04)
1–4	155,456	(73.30)	237,099	(76.06)	392,555	(74.94)
≥5	10,905	(5.14)	20,604	(6.61)	31,509	(6.02)
Visited specialty^a^						
Physician^b^	99,893	(54.14)	138,452	(51.48)	238,345	(52.56)
Surgeon^c^	56,601	(30.68)	72,949	(27.12)	129,550	(28.57)
Others^d^	28,014	(15.18)	57,548	(21.40)	85,562	(18.87)
Types of healthcare facilities^a^						
Long-term care	2,928	(1.58)	6,518	(2.41)	9,446	(2.08)
Primary care	129,480	(69.98)	200,631	(74.33)	330,111	(72.56)
Secondary care	13,695	(7.40)	20,414	(7.56)	34,109	(7.50)
Tertiary care	38,923	(21.04)	42,342	(15.69)	81,265	(17.86)
Residential area^a^						
Urban	94,571	(44.60)	131,528	(42.21)	226,099	(43.17)
Rural	117,488	(55.40)	180,104	(57.79)	297,628	(56.83)
Type of insurance						
National Health Insurance	197,538	(93.14)	276,179	(88.60)	473,717	(90.44)
Medical Aid, Veteran’s Relief	14,544	(6.86)	35,550	(11.40)	50,094	(9.56)
No. of OPD^e^ visits within one year						
0 or 1	191,787	(90.43)	285,795	(91.68)	477,582	(91.17)
2–4	12,254	(5.78)	14,580	(4.68)	26,834	(5.12)
≥5	8,041	(3.79)	11,354	(3.64)	19,395	(3.70)
Comorbidities						
Hypertension	126,438	(59.62)	209,452	(67.19)	335,890	(64.12)
Diabetes mellitus	71,857	(33.88)	104,600	(33.55)	176,457	(33.69)
Hyperlipidemia	71,736	(33.82)	123,920	(39.75)	195,656	(37.35)
Cardiovascular disease (MI^f^ or angina)	31,599	(14.90)	42,366	(13.59)	73,965	(14.12)
Heart failure	22,570	(10.64)	43,549	(13.97)	66,119	(12.62)
Dementia and cognitive impairment	17,334	(8.17)	36,658	(11.76)	53,992	(10.31)
Cerebrovascular disease (TIA^g^)	45,832	(21.61)	69,881	(22.42)	115,713	(22.09)
Peripheral artery disease (PAD^i^)	38,829	(18.31)	65,826	(21.12)	104,655	(19.98)
Chronic kidney disease	6,664	(3.14)	5,847	(1.88)	12,511	(2.39)
Liver cirrhosis	51,766	(24.41)	67,601	(21.69)	119,367	(22.79)
Chronic lung disease (asthma, COPD^h^)	75,575	(35.63)	105,636	(33.89)	181,211	(34.59)
Cancer	39,935	(18.83)	38,203	(12.26)	78,138	(14.92)
Depression	19,635	(9.26)	42,375	(13.59)	62,010	(11.84)
Charlson Comorbidity Index						
0–1	83,991	(39.60)	122,379	(39.26)	206,370	(39.40)
2–4	81,114	(38.25)	129,131	(41.42)	210,245	(40.14)
≥5	46,977	(22.15)	60,219	(19.32)	107,196	(20.46)

^a^Missing data were excluded. Percentages (%) were calculated after excluding the missing data; ^b^Includes: emergency medicine, family medicine, general medicine, internal medicine, neurology, occupational & environmental medicine, pediatrics, psychiatry, rehabilitation medicine, and tuberculosis department; ^c^Includes: cardiothoracic surgery, dermatology, general surgery, neurosurgery, orthopedics, otorhinolaryngology, and urology; ^d^Includes: anesthesiology, dentistry, laboratory medicine, nuclear medicine, oriental medicine, pathology, preventive medicine, radiology, and radiation oncology; ^e^OPD outpatient department; ^f^MI, myocardial infarction; ^g^TIA, transient ischemic attack; ^h^COPD, chronic obstructive pulmonary disease; ^i^PAD, peripheral artery disease

Diagnoses were coded according to the International Statistical Classification of Diseases and Related Health Problems, 10th revision (ICD-10) [23]. Codes for the following comorbidities were collected (see Additional file 1: Table S1): hypertension, diabetes mellitus, hyperlipidemia, cardiovascular disease, heart failure, dementia and cognitive impairment, transient ischemic attack or ischemic stroke, peripheral artery disease, chronic kidney disease, liver cirrhosis, chronic lung disease, systemic cancer, and depression. The Charlson Comorbidity Index [24] was used to estimate the severity of comorbidities of the study population. Polypharmacy was defined as concurrent use of six or more drugs, in accordance with a study in which the potential for inappropriate prescribing has been shown to increase greatly at this threshold [25]. Potential interactions with age, gender, and the Charlson Comorbidity Index (for the number of comorbidities) were explored and none were observed.

### Statistical analysis

Statistical analyses were performed using the SAS software, version 9.3 (Cary, North Carolina). We evaluated subjects’ baseline characteristics using the Student’s *t* test for continuous variables and the *χ*^2^ test for categorical variables. Multivariate logistic regression analysis was used to evaluate risk factors associated with the prescription of PIMs.

## Results

The baseline characteristics of the study population are shown in Table 1. Subjects’ mean age was 73.32 ± 14.44 years, and 59.51 % of subjects were women. Of the 523,811 subjects, a total of 424,064 (80.96 %) were prescribed at least one PIM independent of diagnoses or conditions according to the 2012 Beers Criteria. Most subjects were prescribed one (26.86 %) or two (23.86 %) PIMs; however, the prevalences of three (15.78 %) or four (8.44 %) PIM prescriptions were also high. Table 1 also includes the total number of medications prescribed and the number of medications prescribed from the Beers Criteria. The medications that were not included in the analysis are shown separately (see Additional file 2: Table S2).

Table 2 shows the prevalence of PIM classes that are independent of diagnosis or condition, whereas Table 3 shows the prevalence of PIM classes in specific diagnoses or conditions. The most commonly prescribed medication class independent of diagnosis or condition was first-generation antihistamines with anticholinergic properties (52.33 %). Pain medications (43.04 %) and benzodiazepines (short, intermediate, and long acting; 42.53 %) were next in line. Among the PIM prescriptions considering diagnoses or conditions, subjects diagnosed with delirium (82.21 %), dementia and cognitive impairment (80.24 %) were the most likely to be prescribed PIMs. Elderly patients with heart failure, gastrointestinal disorders, and chronic kidney conditions were also often prescribed PIMs (Table 3).

**Table 2:** Prevalence of prescriptions of potentially inappropriate medications *independent of* diagnosis or condition for older adults (*N* = 523,811)

Drug category		Male (*n* = 212,082) No (%)		Female (*n* = 311,729) No (%)		Total (*n* = 523,81) No (%)
Anticholinergics (excludes TCAs^a^) First-generation antihistamines	105,474	(49.73)	168,642	(54.10)	274,116	(52.33)
Cardiovascular^b^	34,748	(16.38)	13,499	(4.33)	48,247	(9.21)
Central nervous system	102,334	(48.25)	209,620	(67.24)	311,954	(59.55)
Antipsychotics	2,979	(1.40)	4,943	(1.59)	7,922	(1.51)
Tertiary TCAs^c^	11,876	(5.60)	23,220	(7.45)	35,096	(6.70)
Barbiturates	1,043	(0.49)	1,811	(0.58)	2,854	(0.54)
Benzodiazepines	71,452	(33.69)	151,334	(48.55)	222,786	(42.53)
Nonbenzodiazepine hypnotics	14,984	(7.07)	28,312	(9.08)	43,296	(8.27)
Endocrine	6,100	(2.88)	9,128	(2.93)	15,228	(2.91)
Gastrointestinal	18,028	(8.50)	35,961	(11.54)	53,989	(10.31)
Pain	82,101	(38.71)	143,334	(45.98)	225,435	(43.04)
Skeletal muscle relaxants	18,495	(8.72)	36,266	(11.63)	54,761	(10.45)

^a^TCAs, tricyclic antidepressants; ^b^Includes: alpha-agonist (reserpine >0.1 mg/d), alpha-blocker, antiarrhythmic drugs, and digoxin (>0.125 mg/d); ^c^Includes: doxepin >6 mg/d

**Table 3:** Prevalence of prescriptions of potentially inappropriate medications for specific diagnoses or conditions for elderly patients (*N* = 523,811)^a^

Disease or syndrome		Male (*n* = 212,082) No (%^b^)		Female (*n* = 311,729) No (%^b^)		Total (*n* = 523,811)No (%^b^)
Cardiovascular						
Heart failure (*n* = 66,119)	11,624	(17.58)	25,003	(37.82)	36,627	(55.40)
Syncope (*n* = 4,871)	573	(11.76)	509	(10.45)	1,082	(22.21)
Central nervous system						
Chronic seizures or epilepsy (*n* = 16,940)	2,357	(13.91)	3,502	(20.67)	5,859	(34.59)
Delirium (*n* = 3,906)	1,410	(36.10)	1,801	(46.11)	3,211	(82.21)
Dementia and cognitive impairment (*n* = 53,992)	13,557	(25.11)	29,767	(55.13)	43,324	(80.24)
History of falls or fractures (*n* = 76,721)	10,844	(14.13)	34,504	(44.97)	45,348	(59.11)
Insomnia (*n* = 68,919)	7,668	(11.13)	13,876	(20.13)	21,544	(31.26)
Parkinson's disease (*n* = 13,662)	557	(4.08)	1,277	(9.35)	1,834	(13.42)
Gastrointestinal						
Chronic constipation (*n* = 112,212)	26,920	(23.99)	44,320	(39.50)	71,240	(63.49)
History of gastric or duodenal ulcers (*n* = 173,326)	30,178	(17.41)	58,069	(33.50)	88,247	(50.91)
Kidney and urinary tract						
Chronic kidney disease Stages IV and V (*n* = 12,511)	4,153	(33.19)	3,650	(29.17)	7,803	(62.37)
LUTS/BPH^c^ in men (*n* = 68,096)	37,826	(55.55)	-		37,826	(55.55)
Urinary incontinence (all types) in women (*n* = 28,368)	-		118	(0.42)	118	(0.42)
Stress or mixed urinary incontinence (*n* = 6,759)	-		369	(5.46)	369	(5.46)

^a^The prevalences were significantly different between male and female for each diagnosis or condition (*P*<0.0001); ^b^Percentages (%) with respect to male, female, and total populations for each diagnosis or condition, respectively; ^c^LUTS/BPH, lower urinary tract symptoms/benign prostatic hyperplasia

The results of the multivariate regression analysis to identify the factors associated with prescription of PIMs, independent of disease or condition, are presented in Table 4. Female sex (OR = 1.19 and 1.53, respectively), specialties other than physician (that is, surgeon or other; OR = 1.23 and 1.46), severity of comorbidities (OR = 1.21 and 2.25), and polypharmacy (OR = 3.51 and 7.81) were associated factors with PIM prescribing in both subjects with 1–4 PIM and subjects with ≥5 PIM claims. On the other hand, younger age; secondary, tertiary, or long-term healthcare facilities (OR = 0.84 and 0.72); being insured by national health insurance (OR = 0.88 and 0.83); and having not more than one outpatient department visit within the same year (OR = 0.79 and 0.68) showed a decreased association with PIM prescription. Residing in a rural area was positively associated with an increased PIM prescription in subjects with 1–4 PIM claims, whereas in subjects with ≥5 PIM claims, it was negatively associated.

**Table 4:** Multivariate regression analysis for factors associated with prescription of PIM^a^ according to the 2012 Beers Criteria

	Subjects with 1–4 PIM claims	Subjects with ≥5 PIM claims
Characteristics	OR (95% CI)	OR (95% CI)
Age, 65–69 years		
70–74	0.98 (0.89–1.09)	1.16 (1.10–1.22)
75–79	1.16 (1.05–1.29)	1.33 (1.26–1.41)
80–84	1.50 (1.34–1.69)	1.61 (1.51–1.72)
≥85	1.89 (1.64–2.18)	1.93 (1.78–2.09)
Female gender	1.19 (1.17–1.21)	1.53 (1.48–1.59)
Specialty other than physician	1.23 (1.22–1.24)	1.46 (1.43–1.50)
Other than primary care facilities	0.84 (0.83–0.85)	0.72 (0.71–0.73)
Rural area	1.02 (1.00–1.04)	0.85 (0.82–0.88)
Insured by NHI^b^	0.88 (0.85–0.90)	0.83 (0.78–0.88)
No. of OPD^c^ visits within 1 year (≤1 vs. >1)	0.79 (0.78–0.80)	0.68 (0.65–0.71)
Severity of comorbidities^d^	1.21 (1.19–1.22)	2.25 (2.20–2.31)
Polypharmacy^e^	3.51 (3.46–3.56)	7.81 (7.56–8.06)

^a^PIM, potentially inappropriate medication; ^b^NHI, national health insurance; ^c^OPD, outpatient department; ^d^Based on Charlson Comorbidity Index^13^; ^e^Polypharmacy defined as ≥6 medications prescribed in one claim^24^

Charlson Comorbidity Index score and total number of medications were positively correlated with each other (Pearson correlation coefficient = 0.29; *p* < 0.0001). The results of a stratified logistic regression analysis for severity of disease and total number of medications showed that individuals are more likely to have more PIMs if they take a greater number of total medications (see Additional file 3: Table S3).

## Discussion

The results of this population-based study confirmed that PIM prescription is common among elderly Korean subjects: namely, PIM prescriptions independent of diagnosis or condition were found in 80.96 % of prescriptions, while as many as 82.21 % had PIM prescriptions considering specific diagnoses or conditions. This is a much higher rate than those reported in previously published studies from Korea or from other countries [19–21, 25–35]. However, similar results were found in a large outpatient American population, wherein most subjects (80.3 %) filled prescriptions for a single drug from the previously published 2003 Beers Criteria [34], and in a study of Japanese long-term care facilities, wherein 86.5 % of subjects were treated with at least one medication from the 2003 Beers Criteria [27].

One reason for such a high prevalence of PIM prescribing could be the fact that there is no system to inhibit patients from moving from one physician to another, so called “doctor-shopping”, which could have allowed a higher PIM prescription. Moreover, before 2010 when the drug utilization review was introduced in Korea, physicians could not have recognized overlapping prescriptions.

Among the list of medications or medication classes considered inappropriate for any patient 65 years of age and older, the prevalence of benzodiazepines (short, intermediate, and long acting) was high. The fact that benzodiazepines were one of the most commonly prescribed PIM classes was similar to the results of other studies, although the prevalence itself was much higher in our study [20, 30, 35]. Meanwhile, among PIM prescriptions for specific diagnoses or conditions, more than 80 % of subjects diagnosed with delirium, dementia, and cognitive impairment, all of whom must strongly avoid benzodiazepines and anticholinergics [36]—were prescribed at least one PIM.

The high prescription rate of benzodiazepines can be attributed to the high prevalence of insomnia in older adults. Notably, this is associated with increased age, female sex, and the use of benzodiazepines [37–39]. In Korea, older adults with insomnia can easily get benzodiazepine prescriptions from primary care facilities, which do not need to be specialized in psychiatry.

First-generation antihistamines with anticholinergic properties and pain medications were the most commonly prescribed PIMs in this study (Table 2). Antihistamines with anticholinergic properties are often prescribed as supportive medications for common cold, while painkillers are often administered to older adults with musculoskeletal pain (which is mainly due to arthritis). First-generation antihistamines are cheaper than second- or third-generation antihistamines (approximately 1–4 vs. 18–22 US cents per tablet), and nonsteroidal anti-inflammatory drugs (NSAIDs) are cheaper than COX-2 inhibitors (approximately 9–13 vs. 49–57 cents per tablet) [40]; thus, physicians may prefer to prescribe cheaper medications, accounting for the high prevalence of anticholinergics and pain medication prescriptions.

With respect to the risk factors associated with prescription of PIMs independent of disease or condition, female sex [27, 28, 30], severity of comorbidities based on the Charlson Comorbidity Index [24], and polypharmacy [25, 28, 33] were significant risk factors, as has been found in other studies. In subjects with one to four PIM claims, residing in a rural area was associated with an increased risk of PIM prescription, which coincides with the results of a study published by the US Department of Veterans Affairs [41]. In contrast, in subjects with ≥5 PIM claims, living in rural areas seemed to decrease the risk of PIM prescription. We postulated that older adults who received five or more PIMs would have greater comorbidities and the severity of such comorbidities would be higher; as such, they would require treatment in facilities other than primary care (i.e., secondary or tertiary care facilities). In other words, subjects who received five or more PIMs would probably reside in urban areas, where secondary or tertiary care facilities are more commonly located. Thus, it seemed that in this subgroup living in rural areas would decrease the risk of PIM prescription.

The main strength of the present study was the use of a large sample size and a national sample of prescription drug claims, which both allow for generalization of our results to the entire Korean elderly population. Furthermore, we determined PIM prevalence using claim records, which did not depend on subjects’ memory; thus, we were able to avoid recall and selection biases. To our knowledge, this is the first study to evaluate PIM prescription rates in a large outpatient Korean population using the 2012 Beers Criteria.

Nevertheless, there are also some limitations. First, the diagnoses in the KHIRA database may not reflect the actual diagnoses. Therefore, some diseases might be under-diagnosed leading to a lower PIMs prescription estimation. On the contrary, some diseases might have been over-diagnosed causing an overestimation of the PIMs prevalence. However, one report noted that approximately 70 % of discharge diagnoses match the medical records of the hospitals [42]. Second, we analyzed the database of prescription behaviors, and did not collect data on drug-taking behaviors; thus, we cannot be sure that the drugs prescribed in the claims were actually consumed. Third, the 2012 Beers Criteria do not include drug-to-drug interactions or drugs for which dose adjustment is recommended in chronic kidney patients. Some PIMs might have not be included in the analysis due to the lack of this information. Fourth, there may be genetic differences in some drug-metabolizing enzymes [27], which could mean that some drugs classified as PIM according to the Beers criteria, might not suppose a real threat to Korean elderly adults. Fifth, there may be drugs only available in the United States listed in the Beers Criteria and vice-versa — in other words, some drugs that are only available in Korea and thus are not listed in the Beers Criteria may be PIMs for Korean elderly adults. Finally, we lacked detailed clinical information on each subject, meaning that we cannot ignore the fact that, in some circumstances, the use of a PIM may be clinically justifiable if the benefits outweigh the risks to the subject.

Because of the possibility of genetic differences in drug metabolism and because of the different drug lists available in each country, other tools, such as McLeod’s Criteria (1997) [4], the Improving Prescribing in the Elderly Tool (IPET) from Canada (1997) [43], the French consensus panel list (2007) [6], the Screening Tool of Older Person's Prescriptions/Screening Tool to Alert Doctors to Right Treatment (STOPP/START) from Ireland (2008) [7], the Australian prescribing indicators (2008) [8], the Norwegian general practice criteria (2009) [9], the Guideline for Appropriate Drug Use in the Elderly in Korea (2009) [10], and the Korean PIMs list developed in 2010 using the Delphi method [44] might also be needed to properly screen PIM prescriptions. In addition, analyses of drug-to-drug interactions and of dose adjustment for drugs considering impaired kidney function in Korean older adults using the most recently updated 2015 Beers Criteria [45] will be required.

Based on the high prevalence of PIMs prescribing shown in this study, efforts must be made to improve and optimize prescribing behavior in clinical practice. Introducing developed tools and technologies such as drug utilization review and computerized physician order entry with decision support [46] could help reduce PIMs prescription. Furthermore, more clinical and laboratory data are needed to strengthen the sensitivity and specificity of these tools and technologies.

## Conclusions

The results of this study showed that there is a very high prevalence of PIM prescriptions in Korea, which corresponds to the findings of previously published studies. Notably, the prescription rates of benzodiazepines, anticholinergics, and pain medications were the highest. Further development of strategies to reduce PIM prescriptions are needed and additional studies, including reviews of the medical records of subjects with such prescriptions, are also needed to identify the risk factors fostering the high prescription rates of these drugs. Finally, studies to confirm the consequences of prescribing PIMs and the effectiveness of clinical decision supporting systems on healthcare outcomes are also essential.

## Abbreviations

ICD-10, International statistical classification of diseases and related health problems, 10th revision; IPET, improving prescribing in the elderly tool; KHIRA, Korean Health Insurance Review and Assessment; NSAIDs, nonsteroidal anti-inflammatory drugs; PIM, potentially inappropriate medication; START, screening tool to alert doctors to right treatment; STOPP, screening tool of older person’s prescriptions.

## Additional files

Additional file 1: Table S1. A list of comorbidities corresponding to the ICD-10 diagnostic codes. (CSV 8 kb) Additional file 2: Table S2. Drugs excluded from the analysis (unavailable in Korea). (DOCX 17 kb) Additional file 3: Table S3. Stratified logistic regression analysis for the severity of diseases and number of medications prescribed. (DOCX 17 kb)
