# Supplementary material for: Prescription of potentially inappropriate medication in Korean older adults based on 2012 Beers Criteria: a cross-sectional population based study
Source: BMC Geriatr. 2016 Jun 2;16:118. doi: 10.1186/s12877-016-0285-3 (PMC4890525; doi:10.1186/s12877-016-0285-3)
Supplement: Additional file 2: Table S2. — Drugs excluded from the analysis (unavailable in Korea). (DOCX 17 kb) [file 12877_2016_285_MOESM2_ESM.docx]

Additional file 2. Drugs excluded from the analysis (unavailable in Korea)

| Potentially inappropriate medications independent of diagnoses or conditions | |
| --- | --- |
| Dexchlorpheniramine | Amobarbital |
| Clidinium-chlordiazepoxide | Butabarbital |
| Propantheline | Butalbital |
| Guanabenz | Mephobarbital |
| Guanfacine | Quazepam |
| Dofetilide | Meprobamate |
| Dronedarone | Eszopiclone^a^ |
| Ibutilide | Zaleplon^a^ |
| Dronedarone | Methyltestosterone |
| Chlordiazepoxide-amitriptyline^a^ | Desiccated thyroid |
| Perphenazine-amitriptyline^a^ | Glyburide |
| Trimipramine^a^ | Mineral oil, oral |
| Fluphenazine | Meperidine^a^ |
| Asenapine^a^ | Tolmetin^a^ |
| Iloperidone^a^ | Metaxalone |
| Lurasidone^a^ |  |
| Potentially inappropriate medications for specific diagnoses or conditions^b^ | |
| Desipramine |  |
| Protriptyline |  |
| Trimipramine |  |
| Fluphenazine |  |
| Darifenacin |  |
| Hyoscyamine products |  |
| Amphetamine |  |

^a^Also excluded from potentially inappropriate medications for specific diagnoses or conditions; ^b^In addition to the above-mentioned drugs excluded from the criteria for PIM independent of diagnoses or conditions
