# Supplementary material for: Prescription of potentially inappropriate medication in Korean older adults based on 2012 Beers Criteria: a cross-sectional population based study
Source: BMC Geriatr. 2016 Jun 2;16:118. doi: 10.1186/s12877-016-0285-3 (PMC4890525; doi:10.1186/s12877-016-0285-3)
Supplement: Additional file 3: Table S3. — Stratified logistic regression analysis for the severity of diseases and number of medications prescribed. (DOCX 17 kb) [file 12877_2016_285_MOESM3_ESM.docx]

Additional file 3. Stratified logistic regression analysis for the severity of diseases and number of medications prescribed

|  |  | Subjects with 1-4 PIM claims |  | Subjects with ≥5 PIM claims |
| --- | --- | --- | --- | --- |
| Severity of disease | No.of medication prescribed | Unadjusted OR (95% CI) |  | Unadjusted OR (95% CI) |
| 0-1 | 0-5 | 1 |  | 1 |
|  | 6-9 | 2.72 (2.65-2.80) |  | 8.65 (8.42-8.88) |
|  | >=10 | 2.53 (2.39-2.67) |  | 16.07 (15.28-16.90) |
| 2-4 | 0-5 | 1 |  | 1 |
|  | 6-9 | 2.55 (2.43-2.69) |  | 8.72 (8.31-9.16) |
|  | >=10 | 2.66 (2.47-2.87) |  | 18.84 (17.58-20.20) |
| ≥5 | 0-5 | 1 |  | 1 |
|  | 6-9 | 2.81 (2.51-3.14) |  | 11.23 (10.07-12.53) |
|  | >=10 | 2.88 (2.51–3.30) |  | 29.43 (25.87-33.47) |
